# Blood Phenylalanine Levels in Patients with Phenylketonuria from Europe between 2012 and 2018: Is It a Changing Landscape?

**DOI:** 10.3390/nu16132064

**Published:** 2024-06-28

**Authors:** Alex Pinto, Kirsten Ahring, Manuela Ferreira Almeida, Catherine Ashmore, Amaya Bélanger-Quintana, Alberto Burlina, Turgay Coşkun, Anne Daly, Esther van Dam, Ali Dursun, Sharon Evans, François Feillet, Maria Giżewska, Hulya Gökmen-Özel, Mary Hickson, Yteke Hoekstra, Fatma Ilgaz, Richard Jackson, Alicja Leśniak, Christian Loro, Katarzyna Malicka, Michał Patalan, Júlio César Rocha, Serap Sivri, Iris Rodenburg, Francjan van Spronsen, Kamilla Strączek, Ayşegül Tokatli, Anita MacDonald

**Affiliations:** 1 Birmingham Children’s Hospital, Birmingham B4 6NH, UK; catherine.ashmore@nhs.net (C.A.); a.daly3@nhs.net (A.D.); evanss21@me.com (S.E.); anita.macdonald@nhs.net (A.M.); 2 School of Health Professions, Faculty of Health, University of Plymouth, Plymouth PL4 8AA, UK; mary.hickson@plymouth.ac.uk; 3 Departments of Paediatrics and Clinical Genetics, PKU Clinic, Copenhagen University Hospital, Rigshospitalet, Blegdamsvej 9, 2100 Copenhagen, Denmark; kirsten.ahring@regionh.dk; 4 Centro de Genética Médica, Unidade Local de Saúde de Santo António, E.P.E. (ULSSA), 4099-028 Porto, Portugal; manuela.almeida@chporto.min-saude.pt; 5 Centro de Referência na área de Doenças Hereditárias do Metabolismo, Unidade Local de Saúde de Santo António, E.P.E. (ULSSA), 4099-001 Porto, Portugal; 6 Unit for Multidisciplinary Research in Biomedicine, Abel Salazar Institute of Biomedical Sciences, University of Porto-UMIB/ICBAS/UP, 4050-313 Porto, Portugal; 7 Unidad de Enfermedades Metabólicas Congénitas, Hospital Universitario Ramón y Cajal, 28034 Madrid, Spain; amaya.belanger@salud.madrid.org; 8 Division of Inherited Metabolic Diseases, Reference Centre Expanded Newborn Screening, Department of Women’s and Children’s Health, University Hospital, 35128 Padova, Italy; alberto.burlina@unipd.it (A.B.); christian.loro@aopd.veneto.it (C.L.); 9 Division of Pediatric Metabolism, Department of Pediatrics, Faculty of Medicine, Hacettepe University, Gevher Nesibe Cd., 06230 Ankara, Turkey; drturgaycoskun@gmail.com (T.C.); adursun@hacettepe.edu.tr (A.D.); ssivri@hacettepe.edu.tr (S.S.); atokatli@hacettepe.edu.tr (A.T.); 10 Division of Metabolic Diseases, Beatrix Children’s Hospital, University Medical Centre Groningen, University of Groningen, Hanzeplein 1, 9700 RB Groningen, The Netherlands; e.van.dam@umcg.nl (E.v.D.); dietistensectorc@umcg.nl (Y.H.); i.l.rodenburg@umcg.nl (I.R.); f.j.van.spronsen@umcg.nl (F.v.S.); 11 Department of Paediatrics, Reference Center for Inborn Errors of Metabolism, Hôpital d’Enfants Brabois, CHU Nancy, 54500 Vandoeuvre les Nancy, France; f.feillet@chru-nancy.fr; 12 Department of Pediatrics, Endocrinology, Diabetology, Metabolic Diseases and Cardiology, Pomeranian Medical University, 70-204 Szczecin, Poland; maria.gizewska@gmail.com (M.G.); alicjalesniak60@gmail.com (A.L.); chmura.kasia@gmail.com (K.M.); mpatalan@hotmail.com (M.P.); kamillastraczek@wp.pl (K.S.); 13 Department of Nutrition and Dietetics, Faculty of Health Sciences, Hacettepe University, 06100 Ankara, Turkey; hgokmen@hacettepe.edu.tr (H.G.-Ö.); fatma.celik@hacettepe.edu.tr (F.I.); 14 Cancer Research UK Liverpool Cancer Trials Unit, University of Liverpool, Liverpool L69 3GL, UK; r.j.jackson@liverpool.ac.uk; 15 Nutrition and Metabolism, NOVA Medical School (NMS), Faculdade de Ciências Médicas, (FCM), Universidade Nova de Lisboa, 1169-056 Lisboa, Portugal; rochajc@nms.unl.pt; 16 Centro de Investigação em Tecnologias e Serviços de Saúde (CINTESIS), NOVA Medical School (NMS), Faculdade de Ciências Médicas, (FCM), Universidade Nova de Lisboa, 1169-056 Lisboa, Portugal; 17 Reference Centre of Inherited Metabolic Diseases, Unidade Local de Saúde, 1169-045 Lisboa, Portugal; 18 Comprehensive Health Research Centre (CHRC), NOVA Medical School, (NMS), Faculdade de Ciências Médicas (FCM), Universidade Nova de Lisboa, 1169-056 Lisboa, Portugal

**Keywords:** phenylketonuria, phenylalanine, tyrosine, hyperphenylalaninaemia, mild PKU, classical PKU, metabolic control, sapropterin, monitoring

## Abstract

Background: In 2011, a European phenylketonuria (PKU) survey reported that the blood phenylalanine (Phe) levels were well controlled in early life but deteriorated with age. Other studies have shown similar results across the globe. Different target blood Phe levels have been used throughout the years, and, in 2017, the European PKU guidelines defined new targets for blood Phe levels. This study aimed to evaluate blood Phe control in patients with PKU across Europe. Methods: nine centres managing PKU in Europe and Turkey participated. Data were collected retrospectively from medical and dietetic records between 2012 and 2018 on blood Phe levels, PKU severity, and medications. Results: A total of 1323 patients (age range:1–57, 51% male) participated. Patient numbers ranged from 59 to 320 in each centre. The most common phenotype was classical PKU (*n* = 625, 48%), followed by mild PKU (*n* = 357, 27%) and hyperphenylalaninemia (HPA) (*n* = 325, 25%). The mean percentage of blood Phe levels within the target range ranged from 65 ± 54% to 88 ± 49% for all centres. The percentage of Phe levels within the target range declined with increasing age (<2 years: 89%; 2–5 years: 84%; 6–12 years: 73%; 13–18 years: 85%; 19–30 years: 64%; 31–40 years: 59%; and ≥41 years: 40%). The mean blood Phe levels were significantly lower and the percentage within the target range was significantly higher (*p* < 0.001) in patients with HPA (290 ± 325 μmol/L; 96 ± 24%) and mild PKU (365 ± 224 μmol/L; 77 ± 36%) compared to classical PKU (458 ± 350 μmol/L, 54 ± 46%). There was no difference between males and females in the mean blood Phe levels (*p* = 0.939), but the percentage of Phe levels within the target range was higher in females among school-age children (6–12 years; 83% in females vs. 78% in males; *p* = 0.005), adolescents (13–18 years; 62% in females vs. 59% in males; *p* = 0.034) and adults (31–40 years; 65% in females vs. 41% in males; *p* < 0.001 and >41 years; 43% in females vs. 28% in males; *p* < 0.001). Patients treated with sapropterin (*n* = 222) had statistically significantly lower Phe levels compared to diet-only-treated patients (mean 391 ± 334 μmol/L; percentage within target 84 ± 39% vs. 406 ± 334 μmol/L; 73 ± 41%; *p* < 0.001), although a blood Phe mean difference of 15 µmol/L may not be clinically relevant. An increased frequency of blood Phe monitoring was associated with better metabolic control (*p* < 0.05). The mean blood Phe (% Phe levels within target) from blood Phe samples collected weekly was 271 ± 204 μmol/L, (81 ± 33%); for once every 2 weeks, it was 376 ± 262 μmol/L, (78 ± 42%); for once every 4 weeks, it was 426 ± 282 μmol/L, (71 ± 50%); and less than monthly samples, it was 534 ± 468 μmol/L, (70 ± 58%). Conclusions: Overall, blood Phe control deteriorated with age. A higher frequency of blood sampling was associated with better blood Phe control with less variability. The severity of PKU and the available treatments and resources may impact the blood Phe control achieved by each treatment centre.

## 1. Introduction

Phenylketonuria (PKU, OMIM 261600), an inborn error of metabolism, is defined by the dysfunction of phenylalanine hydroxylase (PAH) caused by a defect in the PAH gene. This encodes the hepatic enzyme PAH, which, under standard conditions, catalyses the conversion of the amino acid phenylalanine (Phe) into tyrosine (Tyr), using tetrahydrobiopterin (BH_4_) as a co-substrate. In PKU, if untreated or if therapy is delayed, the toxic accumulation of Phe in the blood, tissue, brain and cerebrospinal fluid is likely to cause severe neurocognitive impairment [1]. Although there is genetic and phenotypical population heterogeneity, the most frequently occurring pathogenic PAH variant worldwide is c.1222C>T (p.Arg408Trp). This is particularly prevalent in Celtic and Eastern European countries and is associated with severe enzyme deficiency [1,2].

Newborn screening detects PKU, and patients treated early are expected to have normal intellectual quotients. As the human brain is intricate and brain complexity continues to increase throughout adulthood, lifelong treatment is essential [3,4]. Blood Phe is the main biomarker used to guide disorder management in PKU. Long-term outcomes and cognition are closely related to blood Phe levels. Recently, in a small group of adults, a strong cognitive improvement was demonstrated when lowering and attaining a stable blood Phe close to 240 μmol/L for 3 to 6 months [5]. Poor metabolic control with elevated blood Phe is associated with cognitive decline and has a negative impact on executive functioning, processing speeds, sustained attention, memory and fine motor control [6]. Inattention may lead to careless mistakes, distraction from tasks, interruption or intruding on others and difficulties in concentrating, organising or completing tasks or following instructions [7]. High blood Phe may also cause ataxia, tremors, clumsiness, headaches, visual loss, anxiety disorders, mood swings and depression [8,9,10,11,12]. Levy et al. reported that almost 70% of adults with PKU had at least one neuropsychiatric comorbidity [13], and neurological deficits may only occur after several years of exposure to high blood Phe levels [14]. In older patients, the risk of comorbidities is higher than in non-PKU controls [15].

Therefore, the maintenance of blood Phe within the therapeutic target ranges is essential to enable optimal neurocognitive outcomes. The European guidelines (2017) recommend a blood Phe therapeutic target range of 120 to 360 μmol/L for patients ≤12 years and between 120 and 600 μmol/L for patients older than 12 years. The United States and Japan’s blood Phe therapeutic target is <360 µmol/L throughout life [16,17,18,19]. Blood Phe control may be affected by many factors including the phenotype severity, target therapeutic blood Phe levels and access to treatment(s) [18].

In PKU, since 2008, there have been considerable advancements in the range of treatments and their delivery, in addition to the introduction of transition and adult clinics. Primary management is dietary Phe restriction supplemented with low-Phe/Phe-free protein substitutes [20]. Special low-protein foods, which accompany Phe restriction, provide energy to promote growth and variety and support dietary adherence. Although the range, quality and palatability of the dietary products have improved (e.g., the introduction of glycomacropeptide as a protein substitute, the increased diversity of special low-protein foods), the global access to low-Phe/Phe-free protein substitutes and special low-protein foods is unequal between countries, potentially affecting patients’ ability to adhere to dietary Phe restriction [21]. In some countries e.g., Poland and Turkey, patients or their families/caregivers are expected to self-fund special low-protein foods at a high cost. However, even when ‘medical foods’ are available on prescription or reimbursed by insurance companies, there may be issues with administration inefficiency, insufficient supplies or poor distribution systems, leading to treatment interruption.

Adjunct pharmaceutical therapies like sapropterin or pegvaliase are treatment options that can improve blood Phe control but are not universally available or appropriate for all patients [2]. Sapropterin dihydrochloride (sapropterin) is a synthetic analogue of tetrahydrobiopterin, the natural co-substrate for the PAH enzyme. It has a chaperone function, stabilising the mutant PAH protein and stimulating residual PAH activity in responsive subjects [2]. Sapropterin, therefore, increases enzyme activity and thereby lowers blood Phe and improves dietary Phe tolerance, although some Phe restriction is usually required. It has also been shown to improve symptoms of inattention, hyperactivity/impulsivity and executive functioning in children and adolescents with PKU [7]. Only around 30 to 40% of patients (with mild phenotypes) respond to sapropterin therapy, and this is defined as a ≥30% reduction in blood Phe from baseline levels [22]. Sapropterin has been approved in Europe since 2008 but has only been reimbursed in the UK since 2021. It is still unavailable in some countries, such as Poland. Data on its long-term use are scarce. Reports from the USA show an improvement in blood Phe over 5 years with patients using sapropterin uninterruptedly [23]. However, blood Phe levels may deteriorate [24], with a high number of patients using sapropterin in the short term only, without attaining the expected benefit [25].

For adults with PKU with uncontrolled blood Phe levels ≥ 600 µmol/L, pegvaliase, an enzyme substitution therapy, is a treatment option. It is a bacterial enzyme that converts Phe into trans-cinnamic acid and ammonia, which are metabolised by the liver and excreted in the urine, and it is given by subcutaneous injection. It is highly effective in lowering the blood Phe levels, with a report from the US indicating that after 3 years of treatment (*n* = 153 patients), the median blood Phe levels had decreased from 1244 to only 167 μmol/L [24]. In addition, some individuals may not require any Phe restriction or supplementation with low-Phe/Phe-free protein substitutes [26]. However, it may take more than 2 years to work effectively, and it is associated with immune-mediated hypersensitivity reactions (commonly injection site reactions and arthralgia), especially during the early months of treatment [27]. Hypophenylalaninaemia may occur with alopecia [26,28], patient anxiety is common when it is first introduced [29], and it requires significant medical supervision. Pegvaliase is approved for adults with PKU in the US (>18 years), the European Union (in 2019, >16 years), Australia, Canada and Japan, but it is expensive, limiting its availability globally [27].

With conventional dietary treatment only, it is well established that a substantial proportion of adult patients treated early maintain blood Phe levels above the recommended target ranges. In 2002, blood Phe control in patients with PKU from four centres (three UK centres and one Australian centre) was reported over a 6-year period from 1994 to 2000 [30]. It was described that 70% of the blood Phe levels were within the target range in patients aged <10 years old, but, among those aged >15 years, only 30% of the blood Phe levels were within the therapeutic target range. A survey from the USA, which enrolled 5530 patients in active follow-up (44 clinics, 52% adults), reported that more than half of the adolescents (13–17 years old) and more than 65% of the adult patients with PKU had unacceptable blood Phe control [31]. Only 33% of the blood Phe levels were within the target range in patients above the age of 30 years. In a further US study, the blood Phe levels of 152 patients (aged 10–40 years, 66% with classical PKU) from two clinics [13] were consistently elevated, with a mean of 584 µmol/L over 5 years. Blood Phe control deteriorated with age.

In Europe, data on blood Phe control are more contradictory. One of the largest European PKU blood Phe audits was conducted in 2010, with the participation of nearly 2000 diet-treated patients with PKU from 10 centres [32]. Data were retrospectively collected over a 1-year period. Although the results were variable between centres, the percentages of blood Phe levels meeting each centre’s local and national target ranges were 88% in children aged up to 1 year, 74% for children aged 1–10 years, 89% for 11–16 years and 65% for adults (>16 years). The frequency of blood sampling declined with age. More recently, a single-centre study by Kanufre et al. [33] showed that less than 30% of patients aged ≥ 12 years on dietary treatment had blood Phe above the local target ranges (<12 years: 120–360 µmol/L; >12 years: 120–480 µmol/L).

The international treatment guidelines for PKU aim to standardise treatment. The first European PKU guidelines, published in 2017, issued 70 recommendations about PKU management [18], including statements about target therapeutic blood Phe levels, the frequency of blood Phe monitoring, treatment management and overall care. With the changing treatment landscape and better management guidance, it is important to establish current blood Phe control across the lifespan in PKU. We aimed to collect data in nine well-established European and Turkish centres, which would indicate whether the current treatment practices have led to metabolic control in line with the first PKU guidelines. These data will provide a baseline to examine whether the European guidelines and new treatments will result in improved metabolic control.

## 2. Materials and Methods

### 2.1. Participating Centres

Clinicians and dietitians from *n* = 7 European and Turkish PKU centres (Ankara, Turkey, Birmingham, UK; Copenhagen, Denmark; Groningen, The Netherlands; Madrid, Spain; Padova, Italy; and Porto, Portugal) who were originally part of the ENEP (European Nutritionist Expert Panel on PKU), and *n* = 2 other centres (Nancy, France; Szczecin, Poland) were invited to participate. All except one centre in the UK (Centre B) provided healthcare for both children and adults. Table 1 shows all participating centres in the study.

### 2.2. Patient Selection

Patients of all ages, with a diagnosis of PKU/hyperphenylalaninemia (HPA), identified by newborn screening and who commenced dietary treatment within the first 3 months of life were included.

Patients were excluded if they were diagnosed late (>3 months) and commenced treatment after 3 months of age and if they had co-existing conditions that may have had an impact on metabolic control (e.g., leukaemia, diabetes), as well as if they were pregnant.

### 2.3. Study Design

This was a multicentre, longitudinal, retrospective study collecting data on the metabolic control of patients with PKU/HPA from 2012 to 2018 inclusively. Data were collected on demographics (e.g., age, ethnicity); mutations (if available, with the exception of Ankara, as their ethical permission excluded mutations); diagnostic Phe levels; blood Phe and Tyr levels; dietary intake (prescribed intake of natural protein, total protein and protein equivalents from protein substitutes); the prescription and doses of pharmaceutical treatments (sapropterin and pegvaliase); anthropometry (height and weight); and the use of vitamin, mineral and energy supplements and other pharmaceutical medications.

### 2.4. Procedures

The PKU PAH deficiency severity was defined by genetic variant analysis in the PAH gene classified by the BIOPKU database [34] or using the diagnostic blood Phe level (HPA, <600 μmol/L; mild PKU (mPKU), 600–1200 μmol/L; classical PKU, >1200 μmol/L) [35]. Data was collected from digital or printed medical and dietetic records by A.P. from 6 centres and collected locally for Centres A, D and F.

This study analysed and described data on overall metabolic control by considering the PKU severity, sex, use of pharmaceutical treatments (e.g., sapropterin) and monitoring frequency. Acceptable metabolic control was categorised using the target blood Phe levels in the European PKU Guidelines 2017 (0–12 y: 120–360 μmol/L; ≥13 y, 120–600 μmol/L). Fasting levels were characterised when the timing of blood samples were available and performed early in the morning.

### 2.5. Statistical Analysis

The sample size was not calculated since all eligible patients being monitored by the centres were invited to participate in this study. The primary outcome of this study was blood Phe control. Continuous data were summarised as the mean ± SD or median (range) depending on normality, whilst categorical data were summarised as the frequency of counts with associated percentages. Heterogeneity analysis was performed. This was defined by estimating the residual SDs, which examined the variability in blood Phe in each patient whilst correcting for any trend that was observed over time. Phe data was analysed using longitudinal regression techniques to take into account the repeated measures. Models fit both the intercept and slope that included random effects. Results were obtained in terms of the mean difference and 95% confidence intervals. Statistical significance was determined by a *p*-value of <0.05 throughout. All statistical analysis was performed by R.J. using the R software (version 3, R Foundation for Statistical Computing, Vienna, Austria).

### 2.6. Ethical Aspects

This project was approved by the individual ethics committees of each centre participating in the study. This study was conducted according to the ‘Declaration of Helsinki’ (52nd WMA General Assembly, Edinburgh, UK, October 2000) and Good Clinical Practice guidelines.

## 3. Results

### 3.1. Treatment Centre Characteristics

Nine European and Turkish PKU centres: *n* = 4 from South/Southeast Europe (Centres A, E, H and I), *n* = 2 from Northern Europe (Centres B and G), *n* = 2 from Western Europe (Centres C and D) and *n* = 1 from Eastern Europe (Centre F) participated in this study (Table 2).

Data were collected from a total of 1323 patients with PKU. Patient numbers ranged from 59 to 320 in each centre. Most of the participants (81%) were recruited from Southern and Northern European centres. The percentage of patients reported as lost to follow-up ranged from 0 in Centres B (paediatric only) and C, to 18% in Centre D. Special low-protein foods (SLPF) were not reimbursed in one centre (Centre F); hence, the patients/parents had to self-pay for these products. In Centre A, only some of the SLPFs were reimbursed, and in Centre C, SLPFs were available through an insurance system only. Sapropterin was not available in Centre F, and Centre B had access via research studies only. Large neutral amino acids (LNAA) were used as a treatment option for adults in Centres A, E, G and I. Only four centres had specialised adult services, only four centres had a psychologist in their multidisciplinary team (Centres C, E, F and I, but Centres C and F only from 2017/18 onwards) and only Centres B and C had a support worker. The number of full-time dietitians dedicated to IMD (PKU) varied. In Centres A and D, dietitians were available, but their time was not dedicated to patients with PKU. All centres except Centre A continued to perform routine blood Phe monitoring during illness/infection.

### 3.2. Subjects’ Characteristics

Table 3 describes the number of patients in each age category, the PKU phenotype and the type of treatment. During the data collection period, the overall mean patient age was 16 years (range 9 to 23 years). Sixty-seven per cent were children and adolescents (infants, *n* = 46, 3%; early/mid-childhood *n* = 567, 43%; adolescents *n* = 268, 20%), and 33% were adults (*n* = 442).

The most common phenotype was classical PKU (*n* = 625, 47%), followed by mPKU (*n* = 357, 27%) and HPA (*n* = 325, 25%), and the remaining 16 patients could not be defined. In 5 of 9 centres, >20% of the patient cohort had HPA. The highest numbers of patients with mPKU were in Centres C and E (47/48%). Classical PKU ranged from 32 to 76% of the patient cohort in each centre, with Centre F having the highest percentage.

Sixty-five per cent of patients were treated with a low-Phe diet alone, while only 17% of patients were prescribed sapropterin with/without dietary treatment. No patients were on pegvaliase treatment.

### 3.3. Blood Phe Control Per Centre

The mean blood Phe levels ranged from 239 μmol/to 391 μmol/L in each centre for all patients studied, with standard deviations ranging from 157 μmol/L to 304 μmol/L (Table 4). The overall percentage of blood Phe levels within the European PKU guidelines’ therapeutic target [18] ranged from 65% to 88% in each centre.

The number of blood spots was highest in Centre B (total *n* = 22,478), with an overall frequency of 33 blood spots per year/per patient, and the lowest frequency was found in Centre A, with three measurements per year/per patient (total *n* = 5838).

### 3.4. Blood Phe and Tyr Levels by Age Group

Table 5 presents data on metabolic control, including the mean blood Phe and Tyr levels and percentage of blood Phe levels within target range for each age group. The mean blood Phe levels increased with age from 187 ± 161 μmol/L in infants (<2 years) to a maximum of 578 ± 474 μmol/L in adults (>41 years). There was also higher variability in the blood Phe levels with increasing age, such that a greater standard deviation was observed from infancy and childhood into adulthood.

A similar trend was observed for the mean percentage of blood Phe levels within the target range. During infancy, the blood Phe levels appeared to be well controlled as the percentage of blood Phe levels within the target range was at its highest (89 ± 39%). This was maintained until the end of adolescence; then, a marked decrease occurred (64 ± 58%). When reaching ≥41 years of age, only 40% of the blood Phe levels met the target levels.

Overall, the mean blood Tyr levels ranged between 58 μmol/L and 68 μmol/L (Table 5).

Data on the mean percentage of blood Phe levels within the target range per age group and centre are presented in Table 6. The number of patients aged <2 years in each centre was low (range: 0–22) but good metabolic control was achieved during this period, except in Centre F, where the mean percentage of blood Phe levels within the target range was only 74 ± 8% (*n* = 2), although the data included diagnostic Phe levels and blood Phe during early sapropterin testing. In preschool children (2–5 years), the mean percentage of Phe levels meeting the target levels lowered to <90% (range: 72–89%). By school age (6–12 years), the mean percentage of blood Phe levels within the target range deteriorated (range: 61–78%) in all centres but two (92 ± 40%, Centre I and 89 ± 31%, Centre C). Both Centres C and I had over 60% of their patient cohort with a combination of mPKU and HPA. Metabolic control improved during adolescence as *n* = 8/9 centres had more than 70% of blood Phe levels below the higher blood Phe target range (range: 73–98%). However, among the eight centres caring for young adults (19–30 years), metabolic control was acceptable in only three centres (Centre C, E and I), where patients were able to maintain at least 75% (range: 75–84%) of their blood Phe levels within the target range. In the remaining centres, the mean percentage of blood Phe within the target range was generally well below 60%. For the older adult age categories, most centres (except Centre C) had less than 40% (as low as 24%) of blood Phe levels within the target range.

### 3.5. Blood Phe Control by Sex

Overall, the mean blood Phe levels were not significantly different in female (*n* = 660) and male (*n* = 663) patients (418 ± 310 μmol/L vs. 389 ± 319 μmol/L, respectively; *p* = 0.939) (Table 7). There was also no difference in the mean percentage of blood Phe levels within the target range between the two groups during infancy (aged 0–5 years; range: 87–92% in females vs. 86–87% in males, *p* > 0.05). However, the number of blood Phe levels meeting the target (%) was higher in females than males in school-age children (6–12 years; 83% in females vs. 78% in males; *p* = 0.005), adolescents (13–18 years; 62% in females vs. 59% in males; *p* = 0.034) and mid-adulthood (31–40 years; 65% in females vs. 41% in males; *p* < 0.001 and >41 years; 43% in females vs. 28% in males; *p* < 0.001). The exception was the age group 19–30 years, whereby the percentage of blood levels in the target range was 44% for females, compared with 47% in males (<0.001).

The mean blood Tyr level was 66 ± 38 μmol/L in females and 68 ± 38 μmol/L in males (*p* = 0.759).

### 3.6. Blood Phe Control by PKU Severity

Data were collected on *n* = 325 patients with HPA, *n* = 357 with mPKU and *n* = 625 with classical PKU. The remaining patients could not be classified regarding their PKU severity due to the lack of data available on mutations or the diagnostic Phe level. The mean blood Phe levels were 290 ± 325 μmol/L in HPA, 365 ± 224 μmol/L in mPKU and 458 ± 350 μmol/L in classical PKU. There was a 36% increase in blood Phe levels in mPKU compared to HPA patients (*p* < 0.001). There was a 25% increase in blood Phe levels in classical compared with mPKU (*p* < 0.001).

The mean blood Tyr level was 71 ± 31 μmol/L in HPA, 64 ± 33 μmol/L in mPKU and 67 ± 41 μmol/L in classical PKU. There was no statistically significant difference between HPA and classical PKU (*p* = 0.279). However, Tyr was lower in mPKU compared with classical PKU (*p* = 0.001).

**Figure 1:**
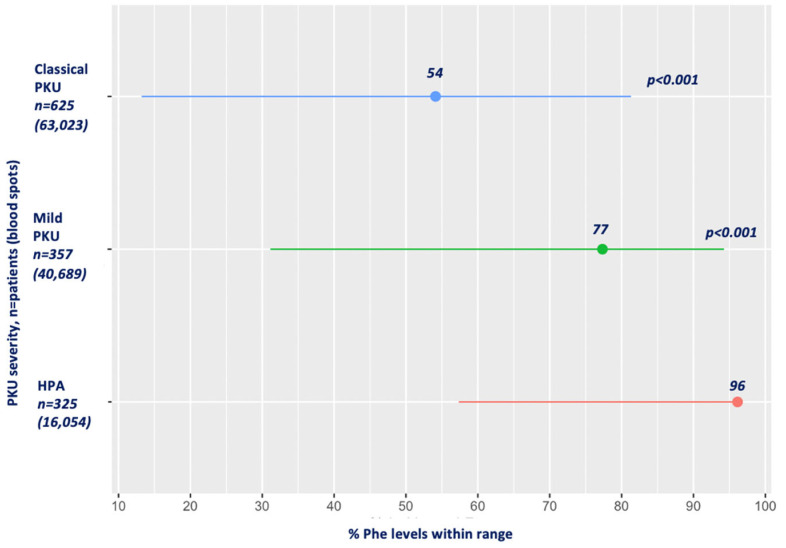
Mean percentage of Phe levels within target range by PKU severity. Abbreviations: Phe, phenylalanine; PKU, phenylketonuria; HPA, hyperphenylalaninemia. Red: HPA; green: mild PKU; blue: classical PKU.

Figure 1 presents the mean percentage of blood Phe levels within the target range for HPA, mPKU and classical PKU. Compared to patients with HPA, in whom 96% of the blood Phe levels were within the target, the mean percentage of Phe levels within the therapeutic target range in both mPKU and classical PKU was significantly lower (77% and 54%, respectively; *p* < 0.001) for all age ranges.

**Figure 2:**
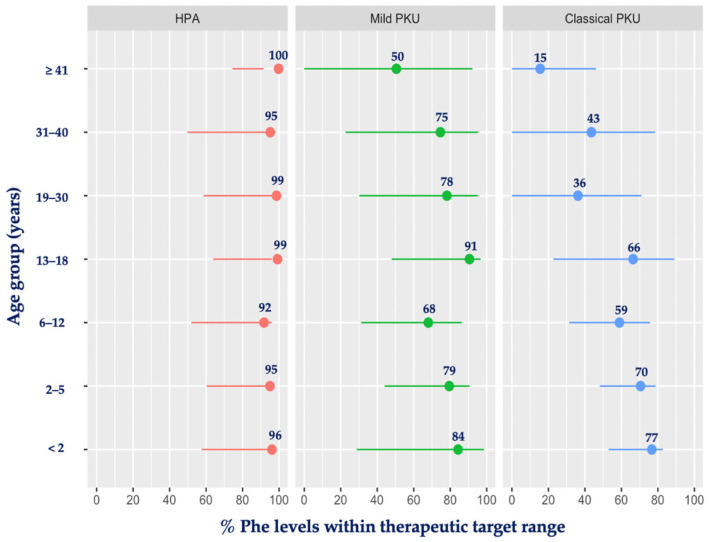
Mean percentage of blood Phe levels within therapeutic target range by age group and PKU severity. Abbreviations: Phe, phenylalanine; PKU, phenylketonuria; HPA, hyperphenylalaninemia. Red: HPA; green: mild PKU; blue: classical PKU.

Figure 2 presents the percentage of blood Phe levels within the therapeutic target range for each level of PKU severity by age group. HPA patients had the majority of their blood Phe levels within the target range, and this was close to 100%. Patients with mPKU had the lowest percentage of levels within the target range when aged ≥ 41 years (50%) and the highest during infancy and adolescence (84% and 91%, respectively). In classical PKU, the mean percentage of blood Phe levels within the target range varied from 59% to 77% during the entire childhood period but was consistently <50% after the age of 18 years, being at its lowest rate at ≥41 years of age, with only 15% of blood Phe levels meeting the therapeutic target range.

### 3.7. Sapropterin and Blood Phe Control

Data on the metabolic control of patients with PKU prescribed sapropterin were only available for *n* = 222 patients (age: 14.6 ± 8.5). The mean blood Phe level in the non-sapropterin group (*n* = 1101; 109,270 blood spots; including all PKU severities) was significantly higher than in the sapropterin group +/− diet (406 ± 334 μmol/L vs. 391 ± 334 μmol/L, respectively; *p* < 0.001). The mean blood Tyr level for the diet-only-treated patients was 68 ± 38 μmol/L, compared with 67 ± 30 μmol/L in the sapropterin group (*p* = 0.217).

**Figure 3:**
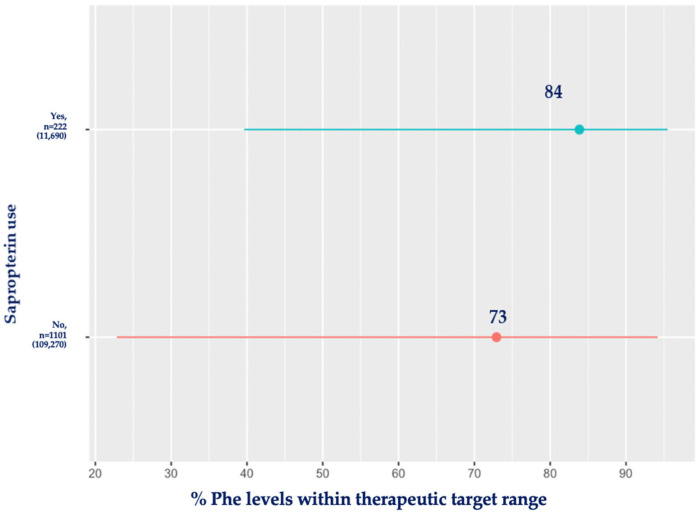
Mean percentage (%) of blood Phe levels within target range for patients using sapropterin compared with diet-only-treated patients. Red: no sapropterin; blue: sapropterin.

Figure 3 compares the percentage of blood Phe within the therapeutic target range for the sapropterin group and the diet-only treatment group (mean time used: 0.9 ± 2 years). Patients using sapropterin maintained 84% of their blood Phe levels within the target range compared to the diet-only-treated group, who had 73% of their blood Phe levels within the recommended ranges (*p* < 0.001).

The comparison of the mean percentage of blood Phe levels within the therapeutic target range in the sapropterin and diet-only treatment groups as analysed by age is shown in Table 8. In the sapropterin group, patients were able to achieve a high percentage of blood Phe levels (≥75%) within the therapeutic target range at all ages (range: 76–93%), particularly during adolescence (13–18 years) and late adulthood (>41 years), with more than 90% of the blood Phe levels meeting the target range. In contrast, in the diet-only-treated group, metabolic control was acceptable until the end of adolescence, as reflected by the high percentage of blood Phe levels within the target (range: 72–89%). However, it deteriorated in adulthood, with only 39–59% of Phe levels within the therapeutic target range.

### 3.8. Blood Phe Variability

In this study, RSD refers to the variability in the Phe levels of each patient, and it is presented by sex, age group, PKU severity and the use of sapropterin in Table 9. The RSD was 319 μmol/L in males, compared to 310 μmol/L in females (*p* < 0.001). It increased with age and was higher in patients with classical PKU. Sapropterin-treated patients had a significantly lower RSD (229 μmol/L vs. 322 μmol/L, *p* < 0.001).

### 3.9. Frequency of Blood Phe Monitoring

The mean blood Phe levels increased with less frequent monitoring, as shown in Table 10. The mean blood Phe levels ranged from 271 μmol/L with weekly blood spots to 534 μmol/L with less than monthly testing (*p* < 0.05). The standard deviation also increased from ±204 μmol/L (weekly blood spots) to ±468 μmol/L (monthly blood spots).

The highest percentage of blood Phe levels within the target range was associated with weekly blood Phe spots (81 ± 33%; *n* = 227; 14,873 blood spots), followed by fortnightly (78% ± 42; *n* = 281; 54,326 blood spots), monthly (71 ± 50%; *n* = 277; 31,599 blood spots) and less than monthly (70 ± 58%; *n* = 538; 20,162 blood spots).

**Figure 4:**
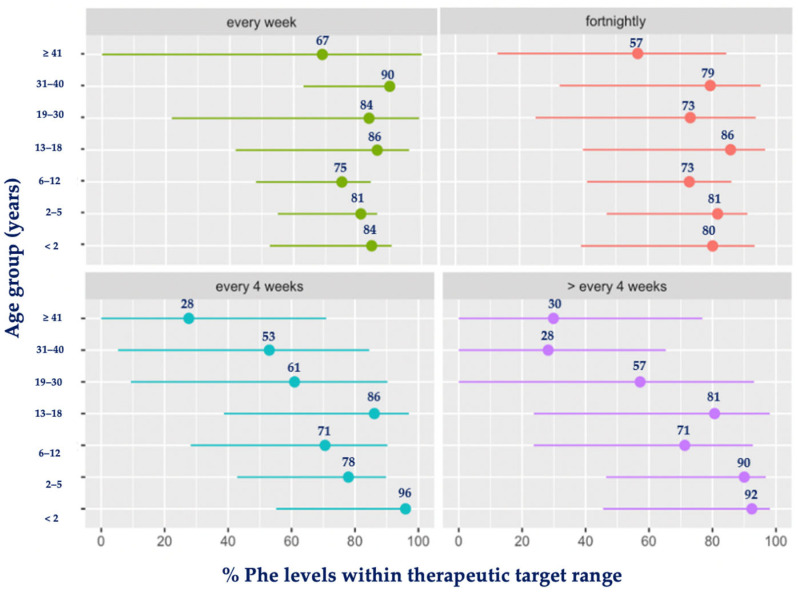
Mean percentage (%) of blood Phe levels within target range by age group and frequency of monitoring. Abbreviations: Phe, phenylalanine. Green: each week; red: fortnightly; blue: every 4 weeks; purple: >every 4 weeks.

Similar results were observed when comparing different age groups (Figure 4). The highest blood Phe levels occurred with increasing age when the frequency of taking blood spots was low.

## 4. Discussion

This retrospective study collected real-world clinical data on a total of 1323 patients with PKU across nine centres in Europe over 7 years from 2012 to 2018 inclusively. When the mean blood Phe level was used as an indicator of metabolic control, in this cohort, the blood Phe achieved was found to be fully in line with the European PKU guidelines for all age groups [18]. However, when examining the percentage of blood Phe levels within the therapeutic target range, the mean percentages varied between 65 and 88% across all centres for the 7-year period observed. Predictably, the blood Phe level, as well as its variability, increased with age, indicating a deterioration in metabolic control, particularly when transitioning from adolescence to adulthood. This is probably associated with an increase in natural protein and the reduced intake of low-Phe/Phe-free protein substitutes. Unsurprisingly, patients with classical PKU had higher mean blood Phe levels and a lower percentage of blood Phe levels within the therapeutic target range compared with patients with HPA and mPKU. Although there was no overall difference in blood Phe levels between female and male patients with PKU, a higher percentage of levels within the target range was found in female patients during school age, adolescence and adulthood, but not in young adults (19–30 years). After the age of 30 years, women had lower mean blood Phe levels when compared with men. Using sapropterin as an adjunct or alternative treatment to the low-Phe diet resulted in significantly lower blood Phe levels. Unlike dietary treatment alone, sapropterin helped patients to sustain acceptable metabolic control post-adolescence. We also report that a higher frequency of blood Phe monitoring improved metabolic control, and this was maintained even with increasing age.

Although, in PKU, it is well established that treatment adherence lowers progressively with age [30,36], we observed that the percentage of blood Phe levels within the therapeutic target range was relatively high (approximately 70–90%) until the age of 18 years, which is similar to earlier studies reported from Europe and USA (Table 11) [13,30,31,32,33,37,38]. However, together with other studies that have reported since the mid-2000s [13,30,31,32,33,37,38], metabolic control did not deteriorate during adolescence, based on the percentage of blood Phe levels within the therapeutic target range. This is probably associated with the relaxation of the European PKU Guidelines target ranges from the age of 12 years (upper limit: 360 μmol/L in <12 years vs. 600 μmol/L in ≥12 years), but, even so, despite the challenges of treatment and adolescence in PKU, the majority were able to maintain their blood Phe within the target range. This finding is encouraging as good metabolic control has been shown to be correlated with both higher IQ and improved executive function in adolescents [39].

Most centres in this study continued to perform blood Phe monitoring during illness, including infection, and this could have contributed to the overall blood Phe control observed. However, illness is usually highest in early childhood and should not explain deteriorating blood Phe control in late childhood and adults.

Considering the emphasis on treatment for life, the introduction of adjunct or alternative pharmaceutical treatments and the increased number of adult care teams, overall blood Phe control in adulthood is disappointing. Using the upper target range of 600 µmol/L, which is higher than in childhood, only around 60% of our adult cohort <40 years of age were able to meet the European PKU guideline on metabolic control. This can be partly explained by the use of a higher upper target range in adults in some of the centres, such as France, Denmark and Spain, during part of the study period (pre-publication of European guidelines), and the French centre continuing to use a higher target blood Phe range following the publication of the European PKU guidelines [18]. Some centres (Turkey, Portugal, Italy and Denmark) used LNAA treatments. Although not recommended by the European guidelines, LNAA competes with Phe at the blood–brain barrier, and, in PKU mice, it has been shown to reduce brain Phe and increase brain non-Phe LNAA and brain monoaminergic neurotransmitter concentrations [40]. These centres permitted a higher blood Phe target for patients prescribed LNAA.

Other factors that may have contributed to the higher blood Phe levels in adults include low risk perception by adult patients [14,41], inconsistent health professional messages about the need for stringent blood Phe control [42,43], low awareness about the importance of lowering blood Phe in adulthood [42], uncomfortable or negative doctor/patient relationships [44] and a lack of access to treatment [45]. Some adult physicians remain unconvinced that adults gain a substantial clinical benefit from better metabolic control [43], and, in some cases, patients have been advised to stop treatment [46].

Although our data showed that blood Phe control deteriorated in adult patients, this may not be a true reflection of the overall landscape in adult PKU care. Our results do not include the blood Phe control of the many adults not being actively followed up within the clinics. In our cohort, some adults with PKU were reported as lost to follow-up (<20%), but this was lower than in previous studies that have reported rates as high as 50% [31,44,47,48]. However, the actual number of patients lost to follow-up was unknown in some of our centres; therefore, this figure may be underestimated and the outcomes of patients not in follow-up is undetermined. It is essential that we strive to gain lifetime knowledge about the blood Phe control of all adult patients with PKU, as it is necessary to establish the impact of blood Phe control on the brain health of early treated patients when they age beyond 60 years of age.

In our cohort, patients on dietary management only or with classical PKU had higher blood Phe variability. The significance of this is not clear, but Romani et al. [49] stated that Phe variation was influential, if not more important than the mean Phe, in predicting adult cognitive outcomes. Hood et al. [50] reported, in 47 school-age children with PKU, that variability in blood Phe was a stronger predictor of cognitive performance than other indices of blood Phe control.

In our PKU centres, clinic resources varied widely, including the availability and reimbursement of SLPFs, pharmaceutical treatments, the number of health professionals and the composition of each multidisciplinary team. In one of our centres (from Poland), which had the highest percentage of patients with classical PKU, SLPFs were not reimbursed, and adjunct drug therapy was unavailable for all patient age groups. This is likely to have adversely affected patients’ ability to be able to adhere to their treatment and ultimately led to less stable blood Phe control. In the US, Jurecki et al. [31] reported that a higher staffing intensity was associated with lower blood Phe levels, particularly in 5- to 12-year-old children and young adults aged 18–29 years. Generally, younger children (0–4 years) and women planning pregnancy receive more attention from their clinic, irrespective of resources [42]. Most centres with psychologists and support workers as part of their multidisciplinary team appeared to have better blood Phe control. However, only four centres had a psychologist and two had a support worker as part of their health professional team. Given the high rates of poor metabolic control in our adult PKU population, it is possible that some patients may have had neuropsychiatric symptoms or lower cognition/executive function that affected their ability to adhere to a Phe-restricted diet. Mental health issues and neurocognitive outcomes should be monitored and supported by neuropsychologists and clinical psychologists as part of routine care [18].

In addition, coordinated transition systems between paediatric and adult clinics, continuous and consistent treatment and regular attendance at clinics is associated with better blood Phe control in adult patients [51] and may prevent loss of follow-up [51,52]. In our study, centres without a structured transition process and an adult metabolic specialist team had higher numbers of patients lost to follow-up. In addition only five of our centres had a transition service in place and three had a partial transition to an adult medical team only, similar to many other European centres. Thus, most adult patients with PKU continue to be treated by paediatricians. As treatment is self-administered and is dependent on the patient’s ability and motivation, good-quality follow-up and monitoring is essential and should lead to better metabolic control. However, in newly established adult clinics, physicians new to the treatment of metabolic disorders, may lead to distrust and low patient confidence.

The poor metabolic control observed in adulthood continues to highlight the shortcomings of the current treatment. Alternative, practical oral therapies associated with minimal side effects are required. Sapropterin was established or introduced in 8 of 9 PKU centres during the data collection period but overall was only prescribed to 222 (17%) patients, even though 52% of the patients either had HPA or mPKU and may have potentially responded to this therapy. Hillert et al. [1] estimated, from a database of 16,092 patients with PKU from 51 countries (22% had mPKU, 16% HPA), that 43% of patients were sapropterin-responsive. Therefore, in our cohort, sapropterin appears to have been under-prescribed. Others have reported the under-utilisation of sapropterin. In a nationwide French study examining the health status of 3549 adults with PKU, only 7% received sapropterin [15]. In our study, the variability in blood Phe levels in the sapropterin group was lower, with better mean blood levels than in the diet-only-treated patients. However, this needs to interpreted with caution given that sapropterin-treated patients have a milder PAH deficiency while non-sapropterin-treated patients might include both milder and more severely PAH-deficient patients. New adjunct therapies are required that are safe and effective for all levels of severity and age groups in PKU.

We studied a mixed cohort of patients with HPA, mild and classical phenotypes, and approximately half of the sample had mPKU or HPA. Overall, the percentage of blood Phe levels within the target range was close to 100% in HPA but decreased in mPKU, and it was only 54% in classical PKU. In general, patients with HPA/mPKU were able to maintain good metabolic control irrespective of their age, but patients with classical PKU struggled to achieve blood Phe levels within target, particularly with increasing age. In our cohort, one third of the centres (Italy, Portugal and Turkey) had a high rate of HPA and mPKU, of which two centres (Italy and Portugal) had a higher percentage of levels within the target range. As patients with milder forms may need less frequent follow-up than patients with classical PKU, specific recommendations should be considered in the future to address the different needs of the diverse phenotypes within this disorder.

The current study found no overall differences in blood Phe levels between males and females (*p* = 0.939). However, in school-age children, adolescents and older adults, females had a higher percentage of blood Phe levels within the target range. It has been suggested that the start of the menstrual cycle could impact the blood Phe levels in girls but there are no data to support this. After the age of 19 y, there was a trend towards a higher percentage of Phe levels within the target in female patients, and their mean blood Phe levels were clinically significantly lower when compared with male patients. There are few data in the literature showing differences between sex, but both Levy et al. [13] and Kanufre et al. [33] found a trend for lower blood Phe levels in female patients over time. This could be associated with women receiving more attention from healthcare professionals from adolescence onwards due to the need to prevent maternal PKU syndrome. In a recent UK survey in adults with PKU, health professionals appeared less concerned about the follow-up of males than females, with clinic visits being less attentive towards male patients and their health [42]. Both males and females should have all of their needs equally met, irrespective of their sex.

The regular monitoring of blood Phe levels is essential in the management of PKU, although the optimal frequency is unknown. The European PKU guidelines suggest less monitoring with increasing age, although this statement does not have a strong evidence base. It is rather counterintuitive to recommend this when it is known that blood Phe control deteriorates with age [18].

Our data indicate that a higher frequency of monitoring of blood Phe levels is associated with better blood Phe control. These results contrast with Jureki et al. [31]. Overall, limited research is available on the benefits of frequent blood Phe sampling. It has been reported that some patients who undergo blood Phe tests infrequently may lower their Phe intake prior to testing, suggesting that their blood Phe levels may be underestimated [53]. Adults may also doubt the necessity for regular blood Phe sampling [53], although, for the parents of children with PKU, regular blood Phe testing may provide some ‘peace of mind’. Patients who are monitored more frequently may be more motivated and their overall treatment adherence is better, leading to less variability in their blood Phe levels, which is important for neurocognitive outcomes [49]. It may also be associated with increased engagement between the patient and health professionals. Some patients perform infrequent blood spots due to problems with blood sampling techniques; they may forget to take blood samples; or they may consider that a lack of blood samples has no effect on the treatment outcome [42].

The availability of blood equipment and the frequency of blood Phe analysis (related to the speed of processing by the lab, postal delays or patients’ frequency of monitoring) may also have an important impact on blood Phe control and treatment adherence. A point-of-care (POC) device capable of measuring blood Phe levels in homes could lower the test costs, provide immediate test results and broaden the accessibility of home Phe monitoring [54]. Currently, there are no approved POC devices, although there has been development over many years. However, it should be remembered that any such device cannot be expected to improve blood Phe control by itself.

### 4.1. Limitations

There are several limitations to our study. We have defined in our study early treatment when diet was introduced within 3 months of life. Since the late 1960s, newborn screening was being introduced and initially resulted in delays in analysis, the reporting of results and the commencement of dietary treatment could be delayed due to the availability of suitable formulas. Data were collected retrospectively from medical and dietetic records. Not all data were available in the different centres, and, in some centres, data were recorded in different source documents and in variable systems. Blood Phe was analysed by nine different laboratories, using different instruments that may have had different sensitivity levels. The Phe from dried blood spots is lower (13% [55] and 7–12% [56]) than that from venous blood samples, suggesting that the actual blood Phe may be higher than what is reported in the literature. Not all centres had data available on the timing of the sample. When this was reported (this was self-recorded by parents/patients so may not reflect the true sampling time), the percentage of fasting samples was as low as 50% in Centre A. It has been shown by MacDonald et al. [57] that the majority of patients with classical PKU on dietary treatment have higher blood Phe levels with early morning fasting. Therefore, in some patients, we may not have achieved a true reflection of their metabolic control. Different target blood Phe levels were used in different countries during the data collection period, especially in older patients, irrespective of the European PKU guidelines. We also included all the blood Phe levels from diagnosis and during the sapropterin loading tests that were conducted, which may have impacted blood Phe control.

Data on mutations and diagnostic Phe levels were also incomplete in some centres. There was some difficulty in defining patient severity due to the ambiguity of some mutations.

Variability in blood Phe levels may be a consequence of less frequent blood Phe sampling, rather than true variability in blood Phe. Statistical significance was identified in some analyses, but given the nature of a large sample size, the differences observed may not all be clinically relevant. We did not collect data on patient comorbidities, patient social backgrounds, economic factors or clinic educational policies.

### 4.2. Strengths

This multicentre study collected data on a large sample size of patients (*n* = 1323) from 9 European clinics. Data were collected on the disorder severity, patient age and frequency of blood Phe sampling in a systematic manner, and the results give clear insights into the impact of each of these factors on metabolic control. Most centres recruited 100% of their PKU clinic population who met the inclusion criteria and gave an accurate reflection of the clinic resources and management strategies used in this group of patients.

## 5. Conclusions

In PKU, overall metabolic control has not improved throughout the years, particularly in adulthood, although more frequent blood Phe sampling and pharmaceutical adjunct therapies appear to have a positive influence on blood Phe levels. The impact of the frequency of blood sampling should be explored in more depth in future studies. Variable and changing blood Phe targets may lead to confusion about the necessity for consistent and lower blood Phe levels in adulthood. In PKU, pharmaceutical therapies are needed that will lower blood Phe levels in all patients without adding to the burden of care.

## Figures and Tables

**Table 1:** Names of participating centres.

Centre	Name of Hospital/Metabolic Centre
A	İhsan Doğramacı Children’s Hospital, Hacettepe University, Ankara, Turkey
B	Birmingham Children’s Hospital, Birmingham, UK
C	Beatrix Children’s Hospital, University of Groningen, University Medical Center, Groningen, Netherlands
D	Department of Pediatrics Hôpital d’Enfants Brabois, CHU Nancy, Vandoeuvre les Nancy, France
E	Centro de Referência na área de Doenças Hereditárias do Metabolismo, Unidade Local de Saúde de Santo António—ULSSA, Porto, Portugal
F	Department of Pediatrics, Endocrinology, Diabetology, Metabolic Diseases and Cardiology of the Developmental Age, Pomeranian Medical University, Szczecin, Poland
G	Department of PKU, Copenhagen University Hospital, Denmark
H	Unidad Enfermedades Metabolicas Servicio de Pediatria Hospital Ramon y Cajal, Madrid, Spain
I	Division of Inherited Metabolic Diseases, University Hospital of Padova, Padova, Italy

**Table 2:** Characteristics of study centres.

	Centre								
	A	B	C	D	E	F	G	H	I
**Number of patients (%)**	320 (24)	97 (7)	101 (8)	96 (7)	116 (9)	59 (4)	314 (24)	62 (5)	158 (12)
**Age group**	Adults and Children	Children Only	Adults and Children	Adults and Children	Adults and Children	Adults and Children	Adults and Children	Adults and Children	Adults and Children
**Number of patients lost to follow-up**	Unknown	0	0	17	4	5	20	5	6
**Reimbursement of SLPFs**	Yes *	Yes	Yes ^+^	Yes	Yes	No	Yes	Yes	Yes
**Reimbursement of protein** **substitutes**	Yes	Yes	Yes	Yes	Yes	Yes	Yes	Yes	Yes
**Reimbursement of sapropterin during study period**	Yes	Limited (Research only)	Yes	Yes	Yes	No	Yes	Yes	Yes
**Home DBS samples sent to the hospital for monitoring**	No (Venous samples)	Yes	Yes	Yes	Yes	Yes	No (Home liquid blood)	Yes	Yes
**Pegvaliase availability**	No	No	No	No	No	No	No	No	No
**Specialist adult team available**	No	Paediatric hospital but patients referred to adult team	Partial ^+^	No	Partial ^+^	No	No	Yes	Yes
**Routine psychologist**	No	No	Yes from 2018	No	Yes	Yes from 2017	No	No	Yes
**Support worker**	No	Yes	Yes	No	No	No	No	No	No
**Number of full-time dietitians dedicated to IMD**	3 (not specific to IMD)	1.3 (specific to PKU)	1.8	5 (not specific to IMD)	2	1	2.7	0	2
**Blood Phe target range (μmol/L)**	**<12 y:** 120–360 **≥12 y:** 120–600	**<12 y:** 120–360 **≥12 y:** 120–600	**<12 y:** 120–360 **≥12 y:** 120–600	**2012–2017:** **<12 y:** 120–600 **≥12 y:** 600–1200 **2018:** **<12 y:** 120–360 **≥12 y:** 360–900	**<12 y:** 120–360 **≥12 y:** 120–480	**<12 y:** 120–360 **≥12 y:** 120–600	**2012–2016:** **0–4 y:** 180–300 **4–8 y:** 180–400 **8–10 y:** 180–600 **10–12 y:** 180–700 **≥12 y:** 180–900 **2018:** **<12 y:** 120–360 **≥12 y:** 360–600	**2012–2016:** **0–6 y:** 120–360 **6–9 y:** 120–540 **10–18 y:** 120–600 **≥18 y:** 120–840 **2018:** **<12 y:** 120–360 **≥12 y:** 360–600	**<12 y:** 120–360 **≥12 y:** 120–600
**Frequency of clinic visits**	**0–1 y:** monthly; **1–2 y:** 2 monthly; **2–3 y:** 3 monthly; **4–18 y:** 4 times a year **>18 y:** 6 monthly	**0–1 y:** 3 monthly; **1–18 y:** 6 monthly	**0–1 y:** 2 monthly; **1–18 y:** 2/3 times a year; **≥18 y:** once a year	**0–1 y:** 3 monthly; **1–18 y:** 6 monthly; **>18 y:** once a year	**0–6 m:** monthly; **6–12 m:** 2 monthly; **1–12 y:** 3 monthly; **12–18 y:** 2/3 times a year; **>18 y:** 1/2 times a year	**0–1 y:** monthly; **2–5 y:** 2 monthly; **6–12 y:** 3/4 monthly; **13–18 y:** 6 monthly; **>18 y:** once a year	**0–6 m:** 2 monthly; **6–18 m:** 3 monthly; **18 m–18 y:** 6 monthly; **>18 y:** once a year	**0–1 y:** monthly; **1–6 y:** 3 monthly; **>6 y:** 6 monthly	**0–1 y:** monthly; **1–12 y:** 6 monthly; **>12 y:** once a year
**Method of blood Phe analysis**	High-performance liquid chromatography	Tandem mass spectrometry	Tandem mass spectrometry	Tandem mass spectrometry	Tandem mass spectrometry	Enzymatic assay for in vitro diagnostic determination of L-phenylalanine in newborn blood spots	Microplate-based enzymatic (PAL)	Tandem mass spectrometry	Tandem mass spectrometry

* Centre A: Not all SLPFs reimbursed; Centre C: SLPFs available through insurance. ^+^ Centre C and E: Adult medical team available but dietitian team remained the same. Abbreviations: *n*, number; SLPFs, special low-protein foods; DBS, dried blood spots; Phe, Phenylalanine; y, years; m, month; IMD, inherited metabolic disorder.

**Table 3:** Mean age and number of patients per age group, PKU phenotype (severity) and type of treatment.

		Centre									Total *n* (%)
		A (*n* = 320)	B (*n* = 97)	C (*n* = 101)	D (*n* = 96)	E (*n* = 116)	F (*n* = 59)	G (*n* = 314)	H (*n* = 62)	I (*n* = 158)	
**Age, years, mean ± SD**		9 ± 6	9 ± 5	23 ± 13	18 ± 13	20 ± 13	11 ± 8	22 ± 13	17 ± 10	14 ± 10	16 ± 10
**Age group,** ***n* of** **patients (%)**	**<2 y**	22 (7)	3 (3)	1 (1)	3 (3)	-	2 (3)	5 (2)	2 (3)	9 (6)	46 (3)
	**2–5 y**	88 (28)	22 (23)	8 (8)	17 (18)	8 (7)	14 (24)	27 (9)	5 (8)	24 (15)	213 (16)
	**6–12 y**	118 (37)	46 (47)	10 (10)	17 (18)	15 (13)	20 (34)	60 (19)	19 (31)	48 (30)	354 (27)
	**13–18 y**	61 (19)	26 (27)	21 (21)	17 (18)	30 (26)	11 (20)	55 (18)	12 (19)	34 (22)	268 (20)
	**19–30 y**	31 (10)	-	33 (33)	23 (24)	53 (46)	12 (19)	86 (27)	15 (24)	28 (18)	280 (21)
	**31–40 y**	-	-	19 (19)	13 (14)	10 (9)	-	43 (14)	9 (15)	13 (8)	107 (8)
	**≥41 y**	-	-	9 (9)	6 (6)	-	-	38 (12)	-	2 (1)	55 (4)
**Phenotype,** ***n* of** **patients (%)**	**HPA**	97 (30)	-	17 (17)	19 (20)	27 (23)	6 (10)	68 (22)	10 (16)	81 (51)	325 (25)
	**mPKU**	69 (22)	38 (42)	46 (46)	21 (22)	54 (47)	8 (14)	82 (26)	13 (21)	26 (16)	357 (27)
	**cPKU**	153 (48)	56 (58)	33 (33)	56 (58)	32 (28)	45 (76)	160 (51)	39 (63)	51 (32)	625 (47)
***n* of patients with** **data on diet (%)**		318 (99)	97 (100)	95 (94)	81 (84)	115 (99)	59 (100)	247 (79)	62 (100)	89 (56)	1163 (88)
**Type of** **treatment,** ***n* of patients (%)**	Diet only *	222 (69)	88 (91)	53 (52)	66 (69)	84 (72)	59 (100)	186 (59)	45 (73)	60 (38)	863 (65)
	**Sapropterin**	39 (12)	9 (9)	42 (42)	15 (16)	19 (16)	-	52 (17)	17 (27)	29 (18)	222 (17)
	**Pegvaliase**	-	-	-	-	-	-	-	-	-	-

Abbreviations: *n*, number; PKU, phenylketonuria; SD, standard deviation; y, years; HPA, hyperphenylalaninaemia; mPKU, mild PKU; cPKU, classical PKU. * Number of patients on diet highly influenced by availability of dietary data (*n* = 160 without data). The majority of HPA patients (*n* = 325) not included in numbers due to lack of assessment.

**Table 4:** Mean blood Phe levels, percentage (%) of blood Phe levels within therapeutic target range and number of blood spots performed per centre.

Centre (*n* of Patients)	*n* of Blood Spots	Overall *n* of Blood Spots Performed Per Patient/Year	% of Levels Performed Fasting * (*n*)	Mean ± SD		
				Blood Phe (μmol/L)	% of Blood Phe within Target Range	Blood Tyr (μmol/L)
^+^ A (*n* = 320)	5838	3	50% (2933)	280 ± 281	70 ± 48	66 ± 40
^+^ B (*n* = 97)	22,478	33	86% (19,242)	239 ± 157	83 ± 30	61 ± 41
C (*n* = 101)	10,038	14	77% (7683)	333 ± 255	79 ± 53	65 ± 32
D (*n* = 96)	6071	9	NA	333 ± 253	67 ± 52	NA
E (*n* = 116)	17,106	21	NA	347 ± 180	87 ± 49	65 ± 29
F (*n* = 59)	8293	20	NA	391 ± 299	66 ± 32	NA
G (*n* = 314)	30,323	14	85% (25,647)	373 ± 273	65 ± 54	76 ± 35
H (*n* = 62)	5540	13	NA	331 ± 283	71 ± 44	NA
I (*n* = 158)	15,273	14	NA	317 ± 220	88 ± 49	61 ± 24
TOTAL	120,960	13	NA	313 ± 304	71 ± 45	66 ± 34

* Blood Phe levels performed fasting was defined when the timing of blood sampling was recorded and it was performed early in the morning. ^+^ Centres A and B have a lower age distribution. Abbreviations: *n*, number; Phe, phenylalanine; Tyr, tyrosine; SD, standard deviation; NA, no data available.

**Table 5:** Mean blood Phe and Tyr levels and percentage (%) of Phe levels within target range per age group.

Age Group	*n* of Patients	*n* of Blood Spots	Mean ± SD		
			Blood Phe (μmol/L)	% of Blood Phe within Target Range	Blood Tyr (μmol/L)
<2 y	46	1618	187 ± 161	89 ± 39	65 ± 31
2–5 y	213	19,164	220 ± 162	84 ± 35	63 ± 38
6–12 y	354	40,553	261 ± 165	73 ± 40	64 ± 43
13–18 y	268	26,901	360 ± 222	85 ± 50	58 ± 39
19–30 y	280	21,501	466 ± 325	64 ± 58	67 ± 41
31–40 y	107	8644	409 ± 322	59 ± 54	62 ± 35
≥41 y	55	2579	578 ± 474	40 ± 60	68 ± 37

Abbreviations: Phe, phenylalanine; *n*, number; y, years; SD, standard deviation; Tyr, tyrosine.

**Table 6:** Mean percentage (%) of blood Phe levels within target range per age group and centre.

Age Group	Mean ± SD, % of Blood Phe Levels within Target Range (*n* of Patients; *n* of Blood Spots)								
	Centre A	Centre B	Centre C	Centre D	Centre E	Centre F	Centre G	Centre H	Centre I
<2 y *	89 ± 38% (22; 247)	96 ± 28% (3; 331)	75 ± 0% (1; 33)	96 ± 50% (3; 35)	NA	74 ± 8% (2; 193)	84 ± 21% (5; 370)	94 ± 50% (2; 114)	95 ± 33% (9; 295)
2–5 y	89 ± 40% (88; 1835)	87 ± 25% (22; 4823)	75 ± 43% (8; 624)	87 ± 32% (17; 1382)	85 ± 43% (8; 1140)	72 ± 11% (14; 2290)	80 ± 18% (27; 4092)	87 ± 19% (5; 517)	88 ± 31% (24; 2461)
6–12 y	61 ± 41% (118; 2654)	78 ± 25% (46; 11,895)	89 ± 31% (10; 800)	76 ± 22% (17; 1843)	73 ± 29% (15; 3070)	68 ± 30% (20; 3630)	75 ± 32% (60; 10,251)	74 ± 38% (19; 2127)	92 ± 40% (48; 4283)
13–18 y	64 ± 50% (61; 787)	84 ± 39% (26; 5429)	78 ± 41% (21; 2344)	77 ± 57% (17; 1327)	98 ± 28% (30; 4966)	73 ± 29% (12; 1566)	86 ± 50% (55; 6217)	85 ± 37% (12; 984)	98 ± 30% (34; 3291)
19–30 y	39 ± 44% (31; 315)	NA	78 ± 42% (33; 3228)	36 ± 52% (23; 614)	84 ± 52% (53; 6630)	51 ± 50% (11; 624)	54 ± 59% (86; 5712)	47 ± 43% (15; 1052)	75 ± 55% (28; 3326)
31–40 y	NA	NA	79 ± 41% (19; 2239)	58 ± 43% (13; 618)	57 ± 49% (10; 1300)	NA	54 ± 57% (43; 2428)	58 ± 49% (9; 746)	34 ± 47% (13; 1313)
≥41 y	NA	NA	64 ± 48% (9; 770)	33 ± 62% (6; 252)	NA	NA	24 ± 51% (38; 1253)	NA	36 ± 26% (2; 304)

* In infants, all blood Phe levels immediately following confirmation of diagnosis and during sapropterin loading tests were included in the analysis. Abbreviations: *n*, number; y, years; NA, not available; SD, standard deviation.

**Table 7:** Mean percentage (%) of blood Phe levels within target range by age group and sex.

Age Group	Female				Male				*p*
	*n* of Patients	*n* of Blood Spots	Mean ± SD		*n* of Patients	*n* of Blood Spots	Mean ± SD		
			Blood Phe	% blood Phe Levels within Target			Blood Phe	% Blood Phe Levels within Target	
<2 y	25	895	134 ± 179	92 ± 27	21	723	179 ± 227	87 ± 33	0.712
2–5 y	97	8483	181 ± 200	87 ± 34	116	10,681	178 ± 203	86 ± 34	0.093
6–12 y	164	16,720	193 ± 202	83 ± 37	190	23,833	234 ± 199	78 ± 42	0.005
13–18 y	131	13,876	324 ± 282	62 ± 48	137	13,025	330 ± 265	59 ± 49	0.034
19–30 y	150	12,375	481 ± 386	44 ± 50	130	9126	412 ± 352	47 ± 50	<0.001
31–40 y	63	5830	319 ± 348	65 ± 48	44	2814	544 ± 457	41 ± 49	<0.001
≥41 y	30	1421	603 ± 539	43 ± 49	25	1158	712 ± 536	28 ± 45	<0.001

Abbreviations: Phe, phenylalanine; SD, standard deviation; *n*, number; y, years.

**Table 8:** Mean percentage of blood Phe levels within target range (%) in patients using sapropterin compared to diet-only-treated patients.

Age Group	% of Blood Phe Levels within Target Range						*p*
	Sapropterin Group			No Sapropterin Group			
	*n* of Patients	*n* of Blood Spots	Mean ± SD	*n* of Patients	*n* of Blood Spots	Mean ± SD	
<2 y	13	227	84 ± 37	33	1667	89 ± 39	0.585
2–5 y	29	755	84 ± 28	184	19,031	84 ± 35	<0.001
6–12 y	60	2164	80 ± 27	294	39,141	72 ± 40	0.419
13–18 y	48	3907	91 ± 47	220	22,352	83 ± 50	<0.001
19–30 y	52	3446	78 ± 45	228	17,691	59 ± 57	<0.001
31–40 y	16	1126	76 ± 44	91	7219	58 ± 53	<0.001
≥41 y	4	65	93 ± 68	51	2169	39 ± 58	<0.001

Abbreviations: Phe, phenylalanine; SD, standard deviation; y, years; *n*, number.

**Table 9:** Estimated residual standard deviation of blood Phe levels by sex, age group, PKU severity and type of treatment.

		Estimated RSD, μmol/L (min; max)	*n* of Patients	*n* of Blood Spots	*p*
**Sex**	Female	310 (0; 6773)	660	59,600	<0.001
	Male	319 (0; 3666)	663	61,360	
**Age group**	<2 y	291 (6; 3240)	46	1618	<0.001
	2–5 y	239 (2; 2993)	213	19,164	<0.001
	6–12 y	201 (0; 6220)	354	40,553	<0.001
	13–18 y	340 (0; 3666)	268	26,901	<0.001
	19–30 y	339 (1; 3000)	280	21,501	<0.001
	31–40 y	362 (6; 2208)	107	8644	<0.001
	≥41 y	510 (2; 2691)	55	2579	<0.001
**PKU severity**	HPA	325 (0; 3666)	325	16,054	<0.001
	Mild PKU	224 (0; 4181)	357	40,689	<0.001
	Classical PKU	350 (0; 6773)	625	64,217	<0.001
**Type of treatment**	No sapropterin	322 (0; 3666)	1101	109,270	<0.001
	Sapropterin	229 (6; 1840)	222	11,690	
	TOTAL	263 (2; 2984)	1323	120,960	

Abbreviations: RSD, residual standard deviation; SD, standard deviation; min, minimum; max, maximum; *n*, number; y, years.

**Table 10:** Mean blood Phe levels according to frequency of blood spot monitoring.

Frequency of Monitoring	*n* of Blood Spots	Blood Phe Level (μmol/L) Mean ± SD	*p*
Weekly	14,873	271 ± 204	-
Once every 2 weeks	54,326	376 ± 262	<0.001
Once every 4 weeks	31,599	426 ± 282	<0.001
>4 weeks	20,162	534 ± 468	<0.001

Abbreviations: Phe, phenylalanine; *n*, number; SD, standard deviation.

**Table 11:** Comparison of blood phenylalanine levels in the literature.

Reference	Country	Data Collection Period	% of Blood Phe Levels within Target Range			Target Blood Phe Levels (μmol/L)	
			Age Group	*n* of Patients	Outcome (%)		
Current study	Turkey, UK, Netherlands, France, Portugal, Poland, Denmark, Spain, Italy	2012–2018	0–1 y: 1–5 y: 6–12 y: 13–18 y: 19–30 y: 31–40 y: ≥41 y:	47 213 353 268 280 107 55	89% 84% 73% 85% 64% 59% 40%	<12 y: 120–360 ≥12 y: 360–600	
Becsei et al., 2022 [37]	Hungary	May 2020–October 2020	2–12 y: >13 y:	51 21	59% (before COVID-19) 51% (during COVID-19) 57% (before COVID-19) 52% (during COVID-19)	<12 y: 120–360 ≥12 y: 360–600	
Kanufre et al., 2021 [33]	Portugal	2017	<12 y: ≥12 y:	19 68	56% 84%	<12 y: 120–360 ≥12 y: 360–600	*(aim for ≤480)*
Levy et al., 2020 [13]	USA	November 2012–November 2017	10–18 y: 19–40 y:	70 82	43% 22%	All ages: 120–360	
Walkowiak et al., 2021 [38]	Poland	2019 and first few months of 2020	0–1 y: 1–2 y: 2–6 y: 6–12 y: 12–18 y: >18 y:	662 548 1140 474 479 933	90% 77% 63% 46% 79% 68%	<12 y: 120–360 ≥12 y: 360–600	*(aim for ≤240)* *(aim for ≤360)*
Jurecki et al., 2014 [31]	USA	July 2014 to September 2015 (variable period, 1 y in total for each patient)	0–4 y: 5–12 y: 13–17 y: 18–29 y: >30 y: >18 y:	637 911 618 660 479 933	88% 72% 78% 61% 79% 49%	All ages: 120–360	
Ahring et al., 2011 [32]	Denmark, Spain Germany, Turkey, Italy, UK, Norway, Poland, Belgium, Netherlands	2007–2008 (1 y in total for each patient)	0–1 y: 1–3 y: 4–10 y: 11–16 y: >16 y:	n/a	88% 74% 74% 89% 65%	0–3 y: 120–360 (*n* = 6 centres) 120–300 (*n* = 1 centre) 42–240 (*n* = 2 centres) 120–400 (*n* = 1 centre) 4–10 y: 120–360 (*n* = 4 centres) 120–400 (*n* = 2 centres) 100–450 (*n* = 1 centre) <480 (*n* = 1 centre) 8–10 y: 120–600 (*n* = 1 centre)	11–16 y: 120–400 (*n* = 1 centre) ≤480 (*n* = 1 centre) 120–600 (*n* = 3 centres) ≤630 (*n* = 1 centre) 100–700 (*n* = 2 centres) <720 (*n* = 1 centre) <900 (*n* = 2 centres) >16 y: 120–400 (*n* = 1 centre) <600 (*n* = 2 centre) <630 (*n* = 1 centre) <700 (*n* = 1 centre) <720 (*n* = 1 centre) <900 (*n* = 3 centres) <1200 (*n* = 1 centre)
Walter et al., 2002 [30]	UK, Australia	1994–2000	0–4 y: 5–9 y: 10–14 y: 15–19 y:	178 137 98 911	72% 73% 50% 21%	<5 y: <360 5–10 y: <480 >10 y: <700	

Abbreviations: Phe, phenylalanine; *n*, number; y, years; n/a, not available; COVID-19, coronavirus disease 2019.

## Data Availability

Data are contained within the article.
